# Highly Crosslinked Agar/Acrylic Acid Hydrogels with Antimicrobial Properties

**DOI:** 10.3390/gels7040183

**Published:** 2021-10-26

**Authors:** Victor H. Pino-Ramos, Lorena Duarte-Peña, Emilio Bucio

**Affiliations:** 1 Departamento de Química Inorgánica y Nuclear, Facultad de Química, Universidad Nacional Autónoma de México, Avenida Universidad 3000, Ciudad Universitaria, Ciudad de México 04510, Mexico; 2 Departamento de Química de Radiaciones y Radioquímica, Instituto de Ciencias Nucleares, Universidad Nacional Autónoma de México, Circuito Exterior, Ciudad Universitaria, Ciudad de México 04510, Mexico; lorena.duarte@correo.nucleares.unam.mx (L.D.-P.); ebucio@nucleares.unam.mx (E.B.)

**Keywords:** biomaterials, hydrogels, biopolymers, antimicrobial material, silver nanoparticles

## Abstract

Hydrogels are three-dimensional soft polymeric materials that can entrap huge amounts of water. They are widely attractive in the biomedicine area because of their outstanding applications such as biosensors, drug delivery vectors, or matrices for cell scaffolds. Generally, the low mechanical strength and fragile structure of the hydrogels limit their feasibility, but this is not the case. In this work, acrylic acid–agar hydrogels with excellent mechanical properties were synthesized using gamma radiation as a crosslinking promoter. The obtained hydrogels exhibited a water absorption capacity up to 6000% in weight without breaking and keeping their shape; additionally, they showed a noticeable adhesion to the skin. The synthesized materials were characterized by infrared spectroscopy (FTIR-ATR), thermogravimetric analysis (TGA), differential scanning calorimetry (DSC), scanning electron microscopy (SEM), and mechanical testing. Additionally, their water uptake capacity and critical pH were studied. *Net*(Agar/AAc) hydrogel exhibited a noticeable capacity to load silver nanoparticles (AgNPs), which endowed it with antimicrobial activity that was demonstrated when challenged against *Escherichia coli* and *methicillin-resistant Staphylococcus aureus* (MRSA) on in vitro conditions.

## 1. Introduction

The biomaterials field has experienced rapid growth in recent decades due to the need to create new polymeric vectors for drug delivery [1], playing an invaluable role in medicine by restoring function and facilitating healing for people after injury or disease [2]. Biomaterials may be natural or synthetic and are widely used in medical applications to support, enhance, or replace damaged tissue or a biological function [2,3]. In this sense, hydrogels have become an important spotlight of study, while it remains a great challenge to obtain hydrogels with excellent mechanical properties, self-adhesion, and resistance to deformation [4,5,6]. These materials have promising applications in biomedicine including, but not limited to, drug delivery, cell scaffolds, sensors, tissue engineering, and wound dressing because they are soft, biocompatible, flexible, and retain huge amounts of fluids or water into their network structure [4,5,6,7]. Hydrogels are commonly used as dressings in the treatment of burns because they absorb the exudate produced by damaged tissue [4,5,6]; besides, if they contain antimicrobial agents, they can act as barriers against tissue infection [7]. Synthesized from natural polymers, hydrogels are generally biocompatible and highly hydrophilic materials, although they present poor mechanical properties [8]; this flaw could be solved by the addition of synthetic polymers such as poly(acrylic acid) (PAAc) or methacrylic acid [9,10], e.g., the combination of synthetic and natural polymers can produce materials that are more resistant to traction and better structured [11]. Besides, the combination of synthetic–natural polymers has attracted attention in recent years due to the feasibility to make new polymers with hybrid or improved properties. Among all the crosslinking methods, employing gamma (γ) radiation is one of the most interesting methods because of its short time of exposition, crosslinking efficiency, sterilizing property, and non-requirement of additional chemical agents [12,13,14,15], ensuring the non-toxicity of the final material [16]. γ-radiation is an eco-friendly tool widely used to sterilize medical devices, food, and cosmetics due to its safety as a sterilizing agent.

PAAc is one of the most studied polymers due to its easy synthesis, water uptake capacity, biocompatibility, pH sensitiveness, and promising applications for drug delivery or sensor manufacturing [1,9,11]. It has been extensively studied for drug delivery vectors in nanogels, hydrogels [17], catheters [18], implants, contact lenses, sensors [19], etc. Besides, PAAc presents adhesiveness that could be useful for topical hydrogels, since it might avoid material accidentally being removed by patient movements [19,20]. Agar is a polysaccharide constituted by a mixture of agarose and agaropectin in different proportions [21]. Agar is composed of a mixture of colloidal polysaccharides of D- and L-galactose (agarose); this biopolymer is industrially important because of its excellent thickening and gelling properties [22,23]; it is extensively studied for the synthesis of hydrogels with applications as a wound-dressing and wound-healing material [21,22,23,24].

In this work, we report a facile method for the synthesis of highly crosslinked hydrogels with excellent mechanical properties obtained by the γ-radiation method using agar and acrylic acid as raw materials and a short reaction time. The hydrogels presented different crosslinking degrees depending on the AAc concentration and the applied dose, which was determined through their excellent mechanical properties. The hydrogels shape was given by the container during the irradiation process; the materials were characterized by several techniques as infrared spectroscopy (FTIR-ATR), nuclear magnetic resonance in solid state (CP/MAS ^13^C-NMR), mechanical testing, differential scanning calorimetry (DSC), and thermogravimetric analysis (TGA). The prepared hydrogels absorbed huge amounts of water without breaking and exhibited a critical pH close to 5. Additionally, they exhibited good antimicrobial activity against *E. coli* and *MRSA* once loaded with silver nanoparticles. Although numerous dressings are already commercially available, there is a great need for the design and synthesis of novel wound-care materials to address the increasing number of burn injuries [25].

## 2. Results and Discussion

**Scheme 1:**
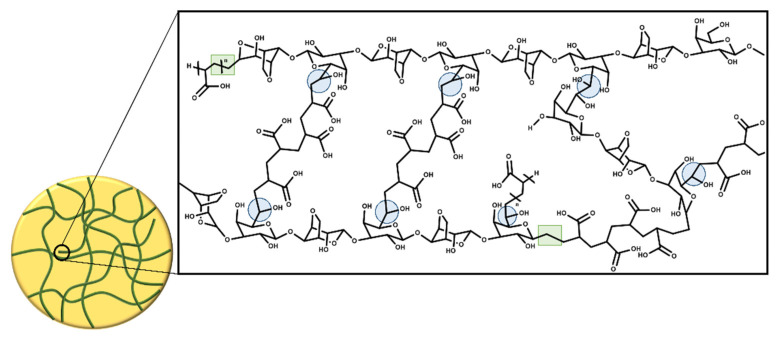
Crosslinking of agar/acrylic acid hydrogels.

The thermal treatment during the addition of AAc to the agar solution is an important part of the synthesis of hydrogels, since during that step, the monomer starts to form oligomers that enhance the density of the solution helping to maintain a jelly form. This jelly consistency made the Petri dishes more handle during the irradiation process, allowing the authors to collocate them at the site of irradiation without so many precautions because the system is not prone to movements. This consistency also facilitated the crosslinking process, since the kinetic motions of the AAc molecules, oligomers, and agar molecules are more restricted than in the liquid state. In this work, the double bond of acrylic acid and the free radicals formed on the agar structure made possible the crosslinking process, so any chemical crosslinker was not used. Scheme 1 shows a representation of a crosslinked hydrogel structure; when the agar is exposed to γ-radiation, free radicals are formed in almost all positions of agar ring except in carbon 1; these free radicals can react with the double bond of acrylic acid, which could lead to the formation of a copolymer [26,27]. However, γ-radiation mainly breaks the C-H bond of C6 of galactose, giving rise to crosslinking of the hydrogel. The formation of this radical occurs in a higher proportion because the C6 is more exposed, since the glycosidic bond does not shield it from radiation [28,29]. The hydrogels were subjected to a solvent extraction process (water) to determine the percentage of gel, and no free polymer was extracted, which means that all polymers had 100% crosslinking agent taking into account AAc as the crosslinking agent. However, the hydrogel can have subsequent crosslinks, as mentioned above, because gamma radiation can form free radicals at other points in the polymer structure, making the hydrogel increasingly rigid. This phenomenon has been observed in many polymers exposed to gamma radiation during their sterilizing process [30].

**Figure 1:**
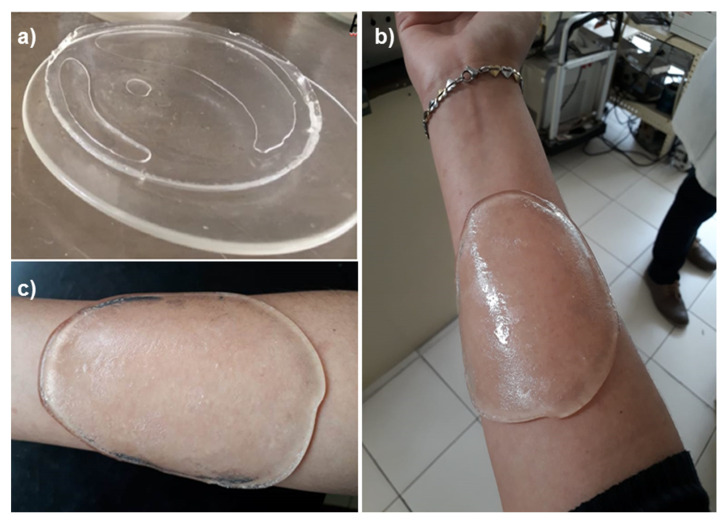
Images of synthesized hydrogels: (**a**) hydrogel freshly washed, (**b**) hydrogel adhered to the skin, (**c**) adhered hydrogel to the skin despite inclination.

The obtained hydrogels were colorless (Figure 1a) and exhibited good flexibility besides good adhesiveness to the skin (Figure 1b,c). These results indicate that γ-radiation is an efficient crosslinker, since it does not involve any chemical agent that reduces the potential biomedical applications of hydrogels; in this method, the hydrogels are sterilized at the same time as being crosslinked.

In the hydrogels synthesis, the crosslinking degree was the total because products after the washing process were not obtained, which is because of the high reactivity that presents the acrylic acid and the applied doses. The agar is susceptible to degradation when exposed to γ-radiation, mainly in the presence of air, so higher irradiation doses were not used; we observed that 20 and 25 kGy were enough to obtain the hydrogels. Regarding the acrylic acid proportions, 30 and 40 vol% of acrylic acid were used to ensure enough molecules that would act as the intermolecular joiners; a lower amount of acrylic acid produces softer hydrogels.

The adhesive property that showed hydrogels to the skin was caused by the presence of carboxylic groups of PAAc on the hydrogel, this behavior has been observed for polyanionic polymers due to their high charge density [19]. The hydrogels’ adhesiveness depended on the crosslinking extent, and the hydrogels that were irradiated at 15 kGy were more adhesive than the ones irradiated at 20 and 25 kGy; this is because the hydrogels obtained at higher irradiation doses did not swell as much as those obtained at 15 kGy, which reduced the interaction of carboxylic groups to the skin.

### 2.1. Characterization

#### 2.1.1. FTIR-ATR Analysis

**Figure 2:**
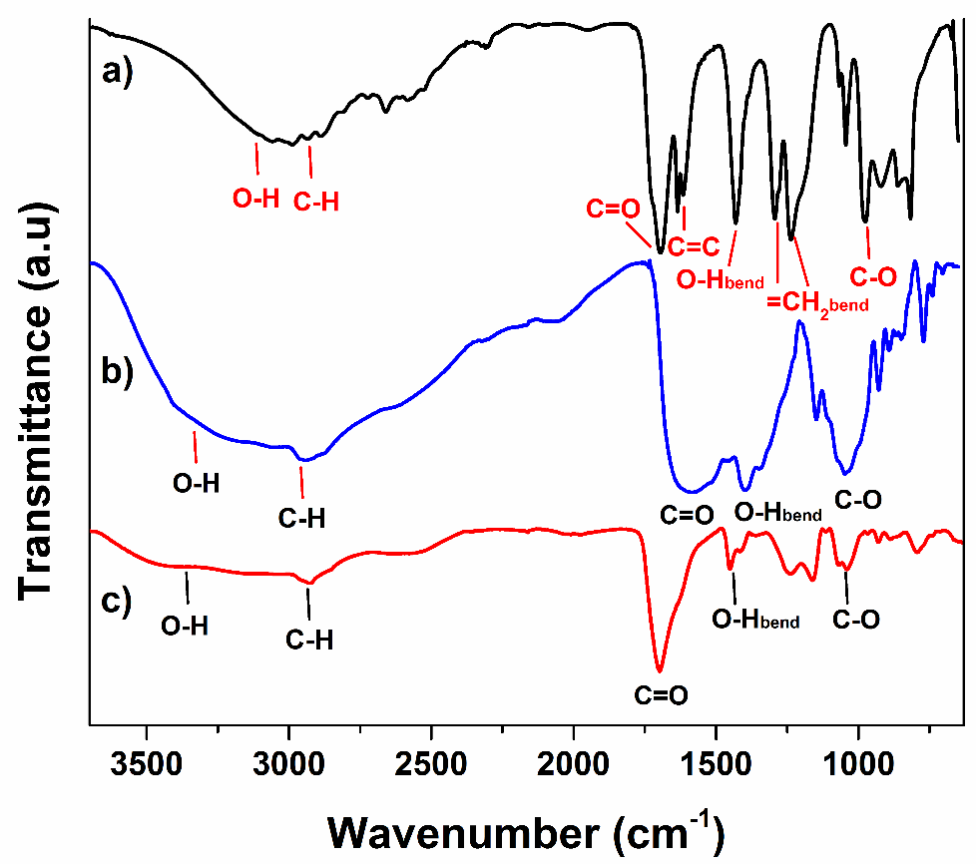
FTIR spectra of (**a**) agar, (**b**) PAAc, and (**c**) hydrogel.

Agar powder presented its characteristic signals according to its structure, the band observed at 3250 cm^−1^ corresponds to hydroxyl groups (O-H) stretching vibration, and the broadness of that peak is because of the presence of different hydroxyl groups on the agar backbone. The signal noticed at 2943 cm^−1^ is associated with sp^3^ C-H stretching vibration; meanwhile, the signal detected at 1581 cm^−1^ belongs to C=O stretching vibrations from the peptide group. Since the agar powder composition contains casein in equal proportion to agar, the peak looks quite broad in that region so the stretching mode of the amine group from casein should also appear, but probably, they are overlapped, since the broad bands start from 1500 cm^−1^ to 1712 cm^−1^ [31]. At 1047, the stretching movement for the C-O bond was observed [32]. Distinctive bands of acrylic acid are observed in Figure 2a. A wideband was noticed for the O-H bond stretching vibrations at 3100 cm^−1^ from the acid group, while the signal for C-H stretching movement of the allyl group was observed at 2988 cm^−1^. The stretching vibration of the carbonyl group appeared at 1695 cm^−1^, next to this band, the stretching vibration signal of the vinyl group at 1634 cm^−1^ was noticed, indicating that the monomer is not homopolymerized (Figure 2b). The signal for O-H bending vibrations from the carboxyl group was noticed at 1431 cm^−1^; the two bands observed at 1294 and 1236 cm^−1^ belong to bending movements of terminal =CH_2_ and stretching vibration of C-O bond of the carboxylic group, respectively [33,34].

Concerning hydrogel, the combination in signals of both materials was observed but mainly exhibited the bands from AAc due to major presence in hydrogel composition (Figure 2c). The main difference between the monomer and hydrogel is the absence of a signal at 1634 cm^−1^ for a vinyl group; this indicates that all the raw material has reacted. The stretching vibrations for the C-H bond and carbonyl group appeared at the same wavenumber, 2943 and 1695 cm^−1^, respectively.

#### 2.1.2. ^13^C-NMR Analysis

**Figure 3:**
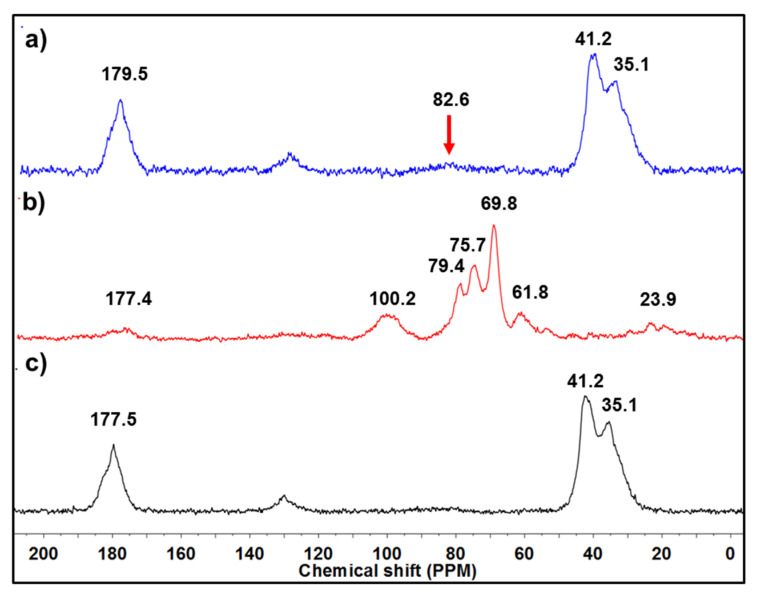
^13^C-NMR spectra of (**a**) hydrogel, (**b**) agar, and (**c**) PAAc.

Figure 3a presents the ^13^C-NMR spectrum of hydrogel, which was analyzed in solid state, as it was not dissolved in any solvents due to its crosslinked structure. The peak observed at 179.5 ppm belongs to the carbonyl group [9], and the signals of 41 and 35 ppm also match with the methylene groups from the reacted acrylic acid; a small peak at 82.6 ppm was observed that might belong to the agar present on the structure of hydrogel. The intensity of peaks that mainly correspond to acidic moiety is because of the proportion used during the synthesis of hydrogel, since the agar was in a solution while the AAc was not. The NMR spectrum of agar exhibited a tiny peak for carbonyl due to the casein present on the composition of agar, which has amide groups; the peak noticed at 100 ppm was assigned to the ether bonds present on the hydrocolloid structure. The peaks observed from 79.4 ppm to 61.8 ppm belong to the carbons that constitute the six-member rings of the backbone. Regarding PAAc, it presented, as we mentioned above, similar chemical shift displacements as hydrogel did (Figure 3c), except by the small peak observed at 82.6 ppm that may indicate the low proportion of agar present on the hydrogel structure.

#### 2.1.3. Scanning Electron Microscopy (SEM)

**Figure 4:**
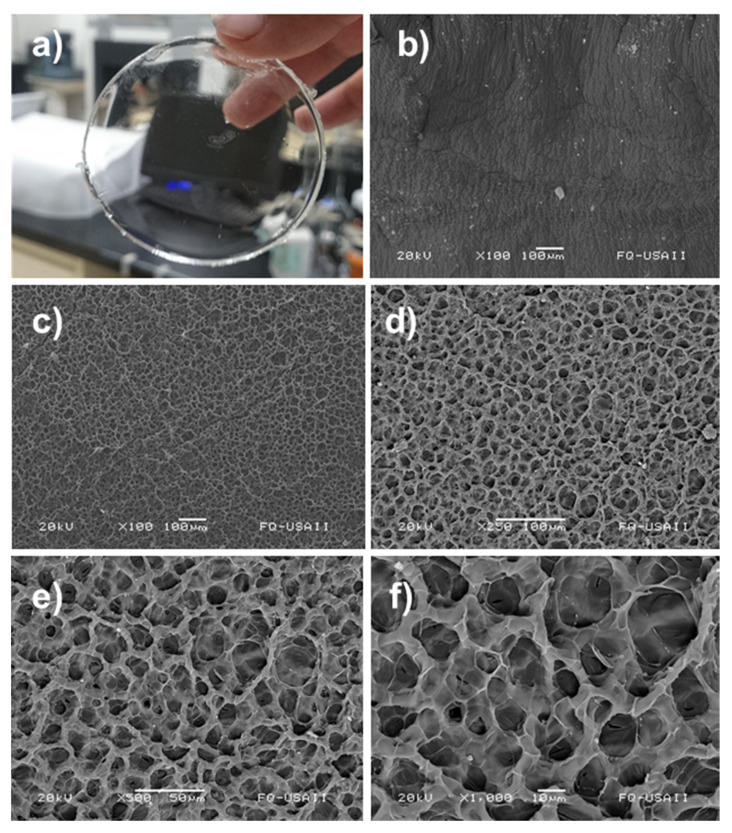
Images of synthesized hydrogel: (**a**) photograph of sample, (**b**) SEM micrograph of hydrogel surface, SEM micrograph of hydrogel cross-section at (**c**) 100×, (**d**) 250×, (**e**) 500×, and (**f**) 1000× magnification, respectively.

The structure of the hydrogels was investigated by SEM analysis; for this essay, the samples were dried by lyophilization. Figure 4a presents an image of hydrogel synthesized in this work, which was colorless and presented a defined circular shape. The dried hydrogel showed a surface with relief (Figure 4b) that morphology could be attributed to the high vacuum to which the material was exposed during lyophilization, since the suction power of the process could cause irregularities in the surface of the hydrogel.

On the other side, the cross-section presented a notable porosity with the pores almost the same size. The pores were formed by the remotion of water during the lyophilization. Figure 4c,d shows the presence of a compact and strong structure with homogeneity in its porosity. Figure 4e,f indicates the absence of small fractions of oligomers or fragments dispersed on the hydrogel; this means that the polymer was highly crosslinked and possible by-products were successfully removed during the washing step.

#### 2.1.4. Thermal Analysis

**Figure 5:**
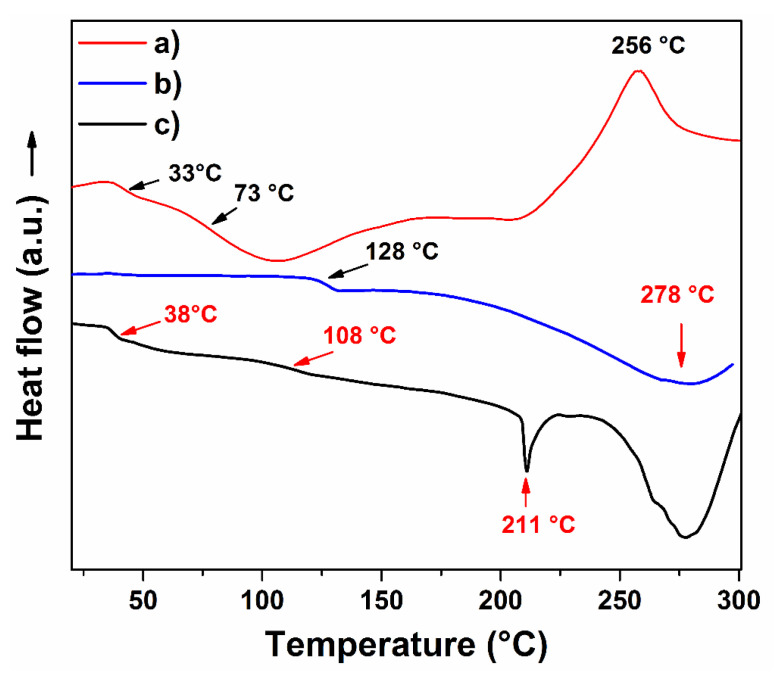
DSC profiles of (**a**) agar, (**b**) PAAc, and (**c**) hydrogel (hydro8).

The thermal behavior of hydrogel was compared with the PAAc and agar powder at the same conditions; this analysis was performed using a heating rate of 5 °C/min to better distinguish thermal transitions; since, when a heating rate of 10 °C/min is used, some weak thermal transitions did not look well defined. Agar exhibited two glass transitions (T_g_); one was observed at 33 °C and another at 73 °C. Those signals are by agarose and agaropectin present on the agar composition. The exothermal signal noticed at 256 °C is associated with the thermal decomposition of the material (Figure 5a). PAAc exhibited a T_g_ of 128 °C and a pronounced endothermic peak at 278 °C (Figure 5b). Concerning hydrogel (hydro8), it displayed a combined thermal profile between PAAc and agar; it presented a T_g_ at 38 °C, as well as two endothermic peaks, one a 211.0 °C and the other at 278.1 °C. The signal noticed at 38 °C is a T_g_ that belongs to PAAc contained on the crosslinked polymer; the peak observed at 108 °C seems to be the T_g_ of PAAc but looks attenuated, this occurred by the combination with agar. The peak of 211 °C was new and can be assigned by the formation of the new polymeric structure by the combination of both materials through crosslinking (Figure 5c). This thermal transition looks similar to a melting point (T_m_); this indicates that the hydrogel presents a compact structure with crystalline domains. The hydrogel thermal profile demonstrated the presence of agar on the hydrogel structure and the obtention of new polymeric material. Additionally, the hydrogel exhibited an enhancement in thermal resistance compared to agar, since no exothermic signals were observed for the hydrogel sample.

**Figure 6:**
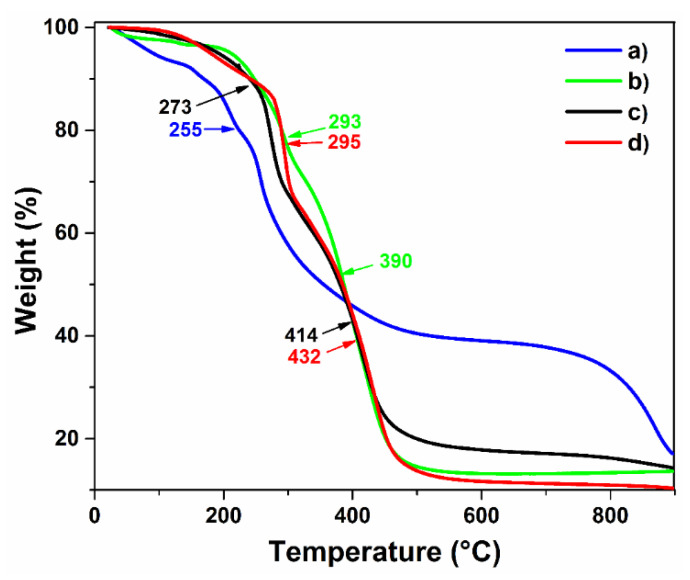
TGA profiles of: (**a**) agar, (**b**) PAAc, (**c**) hydrogel (hydro8), and (**d**) AgNP hydrogel.

Figure 6 presents the thermal stability of the hydrogels compared to raw materials; all samples were studied by thermogravimetric analysis under an inert atmosphere. Agar powder presented an early loss mass before 100 °C, which is attributed to the moisture absorbed for the sample. The decomposition curves noticed at 157 and 209 °C correspond to the dehydration step that underwent agar and the decomposition of agaropectin, respectively. The main decomposition curve observed at 257 °C belongs to the degradation of agarose; it is the main component of agar around 80%, which is the reason that curve was more pronounced (Figure 6a). Finally, the last decomposition curve was observed at 856 °C. On the other hand, PAAc presented a loss mass at 130 °C, which belongs to dehydration of the polymer because it is quite a hydrophilic polymer that can absorb moisture from the air; the loss of this humidity until 130 °C was due to the high affinity to carboxylic groups. At 293 °C, the decarboxylation step of the polymer was observed; at 394 °C, the main thermal degradation of the PAAc backbone was noticed [35].

Concerning hydrogel, the loss mass noticed at 120 °C is due to the loss of absorbed moisture; the degradation curve at 181 °C is associated with the decarboxylation process, and the signal that appeared at 273 °C belongs to the decarboxylation of the PAAc present on the material; meanwhile, at 415 °C, the degradation backbone of the hydrogel was noticed. In general, the thermal profile of hydrogel indicated better thermal stability than agar (Figure 6c). The sample loaded with silver nanoparticles exhibited no remarkable changes compared to the non-loaded sample except for the degradation step at 432 °C, in which a sharper degradation step of the hydrogel was noticed, reaching almost at the total degradation of the sample. This phenomenon might be caused by the presence of metallic nanoparticles dispersed on the polymer, which absorbed the applied heat during the analysis and accelerated the calcination process.

#### 2.1.5. Mechanical Properties

The mechanical test results of the hydrogels are summarized in Table 1. The samples were prepared using different volumes of acrylic acid and irradiation doses. Hydrogels irradiated at 15 kGy using 1 mL of AAc (hydro1) were the samples with the highest elongation due to the applied energy that formed low crosslinking points in the hydrogel structure; however, the hydrogels synthesized under these conditions also exhibited a defined shape and were not dissolved under stirring in water, indicating that the crosslinking degree was enough to remain joined to the structure. The elongation percent decreased as a function of the applied dose due to higher irradiation doses; the crosslinking extent improves due to the formation of a higher number of free radicals that form new intermolecular bonds. As the proportion of AAc used increases, the tensile stress also enhances in hydrogels; this means that materials become more rigid because of the high crosslinking degree in the polymeric system; for example, the samples irradiated at the same dose (20 kGy) but a different proportion of AAc exhibited Young’s modulus of 0.63, 1.2, and 3 megapascals (MPa) for samples with 1 mL, 1.5, and 2 mL of AAc, respectively. Hydrogels synthesized with 2 mL of AAc were those that presented more resistance to traction compared to those obtained with 1 and 1.5 mL of monomer; this indicates that the acrylic acid is responsible for establishing intermolecular connections between the agar backbone and itself; this is through its highly reactive vinyl group. Indeed, hydro7, hydro8, and hydro9 presented an even higher Young’s modulus than reported for silicone rubber film from Goodfellow, which is 1.8 MPa [36]; this comparison confirms the elastomeric behavior of hydrogels.

**Figure 7:**
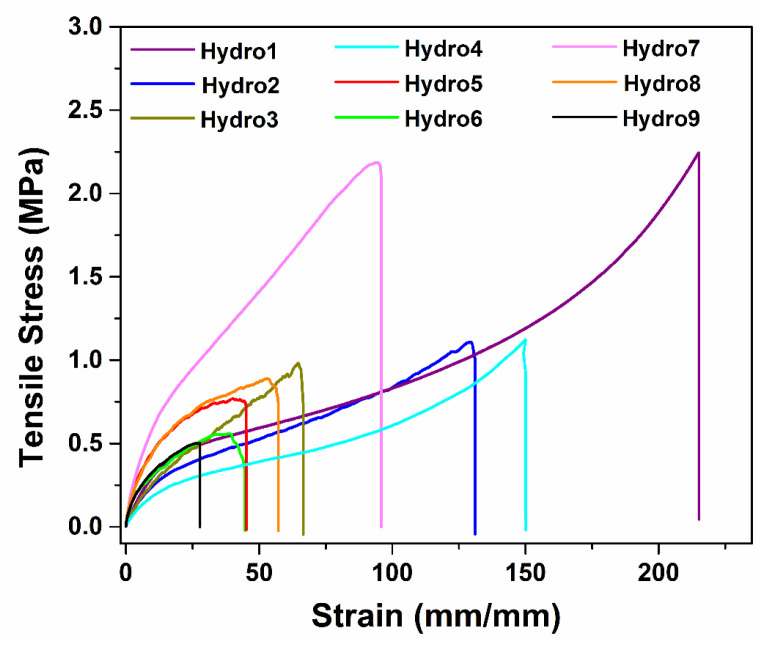
Mechanical properties of synthesized hydrogels.

The irradiation dose was another factor that had a positive effect on the crosslinking extent of hydrogels, since at higher doses, a greater number of free radicals are formed, which are promoters of new bond formation, causing the obtention of more rigid and stable three-dimensional structures. The hydrogels obtained at 15 kGy were the samples that exhibited low resistance to elongation, presenting the highest values of displacement. Hydrogels obtained by applying 20 kGy exhibited minor elasticity compared to 15 kGy, indicating that the stiffness of the hydrogels increased by increment in applied radiation, as was expected; Young’s modulus gradually increased for these samples as a function of the radiation dose; the values were 1, 1.2, and 1.9 MPa for samples irradiated at 15, 20, and 25 kGy, respectively, when the amount of AAc was constant. At 25 kGy, the samples exhibited an even stronger structure than that obtained at 20 kGy; the elongation at the break decreased notably as a function of the dose; although, Young’s modulus was the same for samples obtained with 1 and 1.5 mL AAC. In summary, all the samples presented good elastomeric behavior, and a notable dependence was observed in the amount of AAc and the irradiation dose applied on Young’s modulus (Figure 7).

### 2.2. Swelling Studies

One of the most distinctive properties that hydrogels have is their high capacity to absorb fluids or water due to the high number of hydrophilic groups contained in their structure.

**Figure 8:**
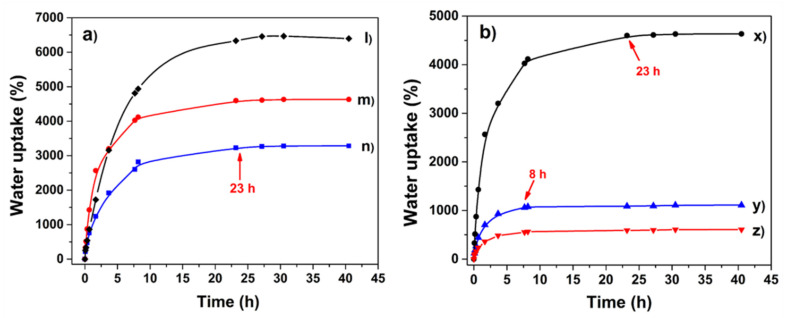
(**a**). Swelling profile of hydrogels obtained at different irradiation dose (l) 15, (m) 20, and (n) 25 kGy, respectively, using 1 mL of AAc. (**b**). Swelling profile of hydrogels as a function of AAc amount (x) 1 mL, (y) 1.5 mL, and (z) 2 mL, irradiated at 20 kGy.

Figure 8a shows the water uptake capacity of hydrogels obtained at different irradiation doses; the irradiation dose was the physical crosslinker used in this work, and its importance is remarkable as noticed in that figure. While the sample obtained by using 15 kGy exhibited an absorption capacity of 6300% by weight, the samples synthesized at 20 and 25 kGy showed 4600% and 3200% of water uptake capacity by hydrogel weight, respectively. These results indicate that the crosslinking extent increases with the increment of the applied doses. Besides, it means that at higher crosslinking degrees, the swelling is lower. Although there is a similar number of hydrophilic groups on the polymer, the joining points impede the expansion of the hydrogel reducing as a result of its water absorption capacity [37]. Despite differences in water absorption, the hydrogels showed the same swelling time (23 h); this likely indicates that hydrogels had a similar porosity and the solvent diffused at the same velocity into the polymer chains.

The results related to the effect of AAc on the swelling of hydrogels are contained in Figure 8b; the amount of AAc dramatically affects the water absorption ability of hydrogels; meanwhile, the sample obtained with 1 mL of AAc retained 4600% of water by weight, and the hydrogel crosslinked with 2 mL of monomer absorbed 600%; although this value is the lowest compared to all the samples, it has a non-negligible absorption capacity. This confirms that AAc is the functional group that makes possible the intermolecular connections in the hydrogel through its double bond, which is very reactive. The maximum swelling time also showed a dependence on the amount of AAc, e.g., hydrogel synthesized with 1 mL of AAc reached its equilibrium after 23 h, while the one obtained with 2 mL reached it in 8 h. Due to the huge quantity of hydrogen bonds and electrostatic interactions present on hydrogel ((l) in Figure 8a), the equilibrium was reached after 23 h since the solvent had to break the inter-chain interactions, which caused the slow polymer expansion. The swelling time was shorter for the hydrogel obtained with 2 mL of AAc (8 h) because this sample had a more rigid structure, which facilitated the solvent diffusion into the polymer ((z) in Figure 8b); besides, this hydrogel does not expand too much due to its high crosslinking degree. In conclusion, the relation between the degree of crosslink and the swelling percentage of samples matches with that observed in the mechanical properties, since hydrogels with a high Young’s modulus presented more stiffness, and in this study, they presented minor water absorption.

### 2.3. Critical pH

**Figure 9:**
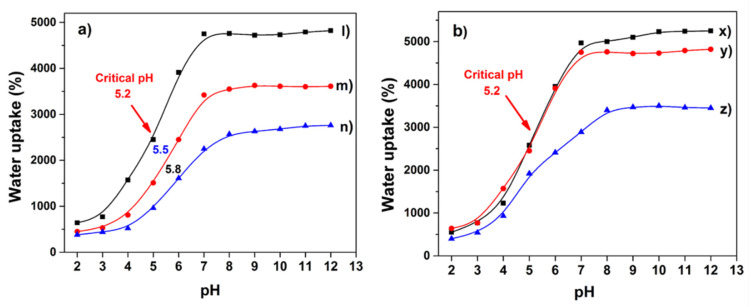
(**a**)**.** Critical pH of hydrogels in PBS as a function of AAc amount (l) 1, (m) 1.5, and (n) 2 mL, irradiated at 20 kGy. (**b**). Swelling profile of hydrogels obtained at different irradiation dose (x) 15, (y) 20, and (z) 25 kGy, respectively, using 1 mL of AAc for these samples.

The hydrogels exhibited notable changes in swelling degree as a function of buffer solution pH. In Figure 9a, it is easily observed that all the samples presented a phase transition; however, the inflection point varied with the AAc content. Hydrogel synthesized with 1 mL of monomer displayed a critical pH of 5.2; for samples obtained with 1.5 and 2 mL of that monomer, they presented values of 5.5 and 5.8, respectively. The samples with higher AAc content presented a critical pH shifted to more basic values. This shift is due to these samples having carboxyl groups with greater closeness, which establishes electrostatic interactions, hydrogen bonds, and Van der Waals between the chains, strengthening the polymer structure [38] and limiting the buffer diffusion into the hydrogel. The water uptake percentage of hydrogels with differences in acrylic acid content is explained in Section 2.2. The response to basic solutions is because of electrostatic repulsions between neighboring deprotonated carboxylic groups. Charge repulsion leads to an expansion in the overall dimensions of the polymer containing the groups [39]. Hydrogels presented a similar electrolytic behavior, despite different applied radiation doses (Figure 9b); they showed the same critical pH value, 5.2, as they have the same amount of ionizable groups. However, this value differs from that commonly observed for PAAc of 4.6 [40]; this signifies that the inclusion of agar causes a slight displacement in pH of the pH-sensitive polymer. This property can be used to control the desorption of a drug or nanoparticle previously loaded on the hydrogel through swell-contraction kinetics [10].

### 2.4. Antimicrobial Activity

The AgNPs’ suspension was brownish, and the average size of AgNPs was 49.7 nm, as is noticed in Figure S1; however, the suspension exhibited two main nanoparticle populations. The major population showed a size of 76 nm and the other one of 9.5 nm, which represented 97% of the total population, 85.2% and 11.8%, respectively. Agglomeration is a phenomenon that is always present in nanometric systems, which justifies the presence of particles of 5 µm size, a small population that represented the 3% particles total. The hydrogel irradiated at 15 kGy (hydro1) almost had no affinity for loading AgNPs, after 24 h of immersion in the nanoparticle suspension, since it retained barely 0.01 mg. On the other hand, the hydrogel obtained at 20 kGy (hydro2) loaded 0.4 mg of AgNPs, indicating that a crosslinking increase improves the interaction between the polymer and the metallic nanoparticles. Hydrogel synthesized at 25 kGy (hydro3) loaded 0.45 mg of silver nanoparticles; this phenomenon can be explained by the crosslinking degree of the materials. As we can see, the hydrogels with the higher crosslinking extent were the ones that retained more AgNPs; this is due to the fact that the carboxyl groups in the 20 and 25 kGy samples are closer to each other and they surround the silver nanoparticle acting as ligands for the metal, while the 15 kGy sample has the carboxyl groups very separated and cannot carry out the cooperative effect to retain the nanoparticle.

**Figure 10:**
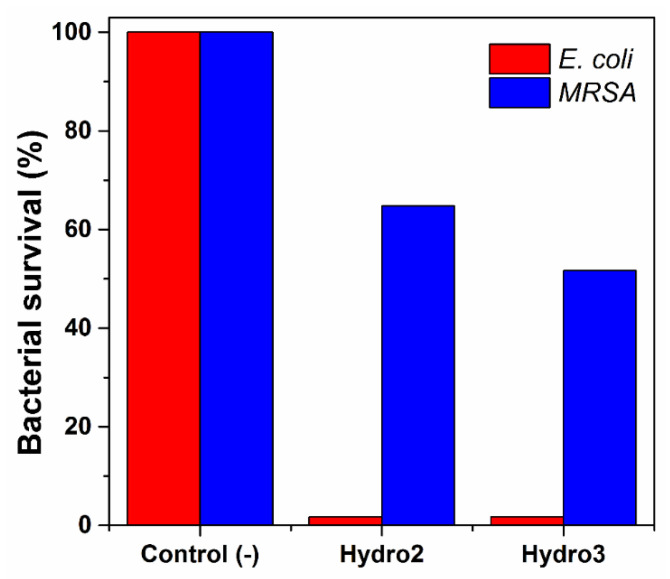
Antimicrobial activity of hydrogels challenged against *E. coli* and *S. aureus* strains.

The hydrogels previously loaded with AgNPs were challenged against bacterial solutions of E. coli and S. aureus. Both samples showed excellent inhibitory activity against E. coli; they eradicated almost the whole bacterial population of that strain (Figure 10). However, their effectiveness was lower for methicillin-resistant Staphylococcus aureus; hydro2 reduced close to 45% of bacteria, while Hydro3 eradicated 50% of the MRSA population. This bacteria showed major resistance to silver nanoparticles because of the thickness of its cell wall; Gram-positive bacteria as Staphylococcus aureus possess a thick wall of peptidoglycan that limits the diffusion of external agents into the cell, while E. coli, a Gram-negative bacteria, has a thin layer of lipopolysaccharides in its external membrane and that makes them more susceptible to biocides [41]. The difference in antimicrobial efficacy showed by hydrogels is by the amount of previously loaded AgNPs, since hydro3 had a higher content of AgNPs. All the above mentioned supports the idea that hydrogels might have biomedical applications as wound-dressing materials; besides, several studies have demonstrated the hemocompatibility of the silver nanoparticles [42,43], reinforcing such a hypothesis.

## 3. Conclusions

Highly crosslinked hydrogels with excellent mechanical properties were successfully synthesized by reacting acrylic acid and agar through the γ-radiation method. Usually, hydrogels have poor mechanical strength and low deformation, which unfortunately sets limitations to their high-end applications but not in this work. The synthesized materials exhibited a notable resistance to traction, and Young’s modulus from 0.6 Mpa to 3 MPa depends on the AAc content and/or applied dose; this means that using this method, we can choose how elastic we want the hydrogel to be; besides, the use of a biopolymer such as agar, a highly reactive monomer such as acrylic acid, and γ-radiation such as a crosslinker agent guarantees the biocompatibility of the hydrogel. The hydrogels loaded with silver nanoparticles showed notable antimicrobial activity against *E. coli* and methicillin-resistant *Staphylococcus aureus*. As a consequence of all the above mentioned, synthesized hydrogels could be potentially used in biomedical applications for burn-wound dressing and preventing bacterial infections on the affected skin.

## 4. Materials and Methods

### 4.1. Materials and Methods

Agar (without pretreatment) and acrylic acid (purified before use by vacuum distillation), sodium citrate, silver nitrate, and sodium hydroxide were purchased from Sigma–Aldrich, St. Louis, MI, USA. Citric acid, boric acid, and trisodium phosphate dodecahydrate were acquired from J.T. Baker, Mexico. *E. coli* (ATCC 31165) and S. aureus (ATCC 29213) were provided by the Microbiology Department of Chemistry School of National Autonomous University of Mexico. All the experiments were carried out with bidistilled water.

### 4.2. Preparation of Hydrogels

The hydrogels were prepared by dispersing 1 g of agar in 50 mL of distilled water under magnetic stirring; then, the mixture was heated for a few minutes up to 90 °C to complete dissolution. Thereafter, 5 mL of freshly prepared agar solution were transferred into a tiny beaker keeping a temperature of 60 °C; immediately, a pre-established amount of AAc under constant stirring was added. The obtained solution was stirred for 8 min at that temperature and, then, transferred to Petri dishes and cooled at room temperature. After that, the samples were exposed to different irradiation doses on a ^60^Co γ source Gammabeam 651 PT of Nordion International Inc to obtain hydrogels with different crosslinking degrees; the intensity of the γ-radiation was 12.3 kGy/h.

The obtained hydrogels were washed (3 times) in distilled water under constant stirring to remove the by-products. Finally, the hydrogels were dried through lyophilization for their subsequent characterization and other studies.

### 4.3. Synthesis of AgNPs

An AgNO_3_ solution of 1 × 10^−3^ M concentration was prepared by dissolving 18 mg of the salt in 100 mL of distilled water; the solution was stirred until complete dissolution; then, it was adjusted to pH 8 with a 1 M solution of NaOH. The solution was heated at 85 °C under continuous stirring for 10 min, during this process, 1 mL of sodium citrate solution (1% *w*/*v*) was added. The solution was kept on the heat for one hour at 85 °C; until the solution acquired a brownish color [44]. After that, it was cooled at room temperature, obtaining a suspension of 18 µg/mL. The absorbance of the colloidal system was determined by UV-vis spectroscopy in a Specord 200 plus spectrophotometer (Analytik Jena AG, Jena, Germany).

AgNP size was determined using Malvern Zetasizer Nano S equipment and DTS (nano) software from Malvern instruments, Madrid, Spain. Diluted samples were sonicated for 10 min to deagglomerate the nanoparticles; then, the suspension was placed in disposable polystyrene cuvettes, and the scattering intensity was measured at 25 °C. Water was used as the dispersant. The analysis was carried out by triplicate, and several records were performed in each analysis.

### 4.4. Structural Characterization

Hydrogels were characterized by FTIR-ATR spectroscopy in a Nicolet iS5 Thermo scientific (USA) with 16 scans from 4000 to 650 cm^−1^. The size of synthesized AgNPs was determined by dynamic light scattering using Malvern Zetasizer Nano S, and their shape was determined by scanning electron microscopy (SEM, JEOL JSM-5900, Tokyo, Japan) operating at 200 kV. Solid-state crosspolarization magic angle spinning carbon-13 nuclear magnetic resonance (CP/MAS ^13^C-NMR) was used to analyze the structure of grafted films in a Bruker Avance III HD at 400 MHz. Tensile test specimens of hydrogels were adequately cut for analysis of mechanical properties of samples according to ASTM standards. For this analysis, were used hydrogels freshly prepared; samples were evaluated in an AGS-X-Shimadzu extensometer under the following conditions: 44% moisture, an extensional speed of 10 mm/min at room temperature. The measurements were carried out by triplicate for each sample, and data of a specific sample were plotted.

### 4.5. Thermal Analysis

An amount of 5–10 mg of each sample was encapsulated in a hermetically sealed aluminum pan; then, it was collocated into the oven of a DSC Q100 (TA Instruments, New Castle, DE, USA) equipment. The first heating was carried out in the temperature range of 20 to 100 °C to remove some residual water or moisture, while the second heating cycle was carried out in the temperature range of 25 to 300 °C to observe the T_m_ and T_g_ of the sample. All DSC experiments were carried out in an inert atmosphere (nitrogen), and the heat flow rate was 5 °C/min. On the other hand, the thermogravimetric analysis was carried out in a TGA Q50 (TA Instruments, New Castle, DE, USA) under nitrogen atmosphere; for this, samples from 5 to 15 mg were analyzed using a heating ramp of 10 °C/min from 25 to 900 °C.

### 4.6. Swelling Studies and Critical pH

Pieces of dry hydrogels were weighed and, then, immersed in distilled water at room temperature; the gain of mass was measured gravimetrically at pre-established intervals of time between 15 min until 48 h of immersion. For each measurement, the sample was taken out from the medium and weighed after removing excess water with an absorbent paper. The swelling percentage was calculated using the following equation:

Swelling (%) = [(W_s_ − W_d_)/W_d_] × 100 (1)

where W_s_ and W_d_ are the weights of the swollen and dried pieces, respectively.

Hydrogels were immersed in phosphate buffer solutions (PBS) of different pH, from pH 2 to pH 12. The PBS was prepared by mixing different proportions of boric acid 0.2 M and citric acid 0.05 M solutions with trisodium phosphate dodecahydrate aqueous solution 0.1 M. The hydrogels were weighed once they reached their corresponding maximum water uptake capacity; this was determined taking into account the swelling time previously obtained. After each measurement, the excess water was removed with absorbent paper. The critical pH value was defined as the inflection point in the swelling degree and solution pH plot. The experiment was carried out at room temperature in triplicate.

### 4.7. Load of AgNPs

Hydrogel samples were cut in circles of 1 cm in diameter and were washed in distilled water; then, they were dried in an oven vacuum at 40 °C until they were a constant weight. Once dried, the circles were weighed and collocated in vials with 5 mL of silver nanoparticles (0.018 mg/mL) previously prepared under constant stirring for 24 h at room temperature. The loading process was monitored by changes in absorbance at 411 nm using an Analytikjena Specord 200 Plus spectrophotometer. The amount of loaded NPs was calculated by using calibration curves and the following equation:

AgNPs loaded (mg/g) = (C_1_ − C_2_) × *v*/*w* (2)

where C_1_ and C_2_ represent the initial and final concentration of the solution, respectively; *v* is the volume of solution used and *w* the weight of the sample.

### 4.8. Antimicrobial Assay Using E. coli and MRSA

Hydrogel samples previously loaded with silver nanoparticles were put into a 12-well plate containing E. coli or MRSA bacterial solution of a concentration of 0.5 McFarland (1 × 10^8^ bacteria). A test tube with a bacterial solution was used as a negative control. The plates were incubated at 37 °C for 18 h under aerobic conditions. After the incubation step, 100 µL of each treatment medium was taken and passed through a 96-well plate. The absorbance was measured at 600 nm using a microplate reader (Biotek, Winooski, VT) to determine the percentage of bacterial survival.

## Figures and Tables

**Table 1:** Mechanical properties of hydrogels synthesized at different doses and monomer concentrations.

Sample	Vol. AAc (mL)	Radiation Dose (kGy)	Strain (%)	Strain (mm/mm)	Young’s Modulus (MPa)
Hydro1	1.0	15	860.6	215.0	0.90
Hydro2	1.0	20	524.2	131.0	0.63
Hydro3	1.0	25	423.8	66.6	0.81
Hydro4	1.5	15	589.9	150.2	1.0
Hydro5	1.5	20	155.0	46.0	1.2
Hydro6	1.5	25	360.0	44.6	1.9
Hydro7	2.0	15	383.4	90.9	3.04
Hydro8	2.0	20	268.0	56.1	3.0
Hydro9	2.0	25	180.6	27.6	2.0

## Data Availability

Not applicable.

## Supplementary Materials

The following are available online at https://www.mdpi.com/article/10.3390/gels7040183/s1, Figure S1: DLS measurements of silver nanoparticle suspension.
