# Supplementary material for: Highly Crosslinked Agar/Acrylic Acid Hydrogels with Antimicrobial Properties
Source: Gels. 2021 Oct 26;7(4):183. doi: 10.3390/gels7040183 (PMC8628682; doi:10.3390/gels7040183)
Supplement: Supplementary file 1 [file gels-07-00183-s001.zip › gels-1409219-supplementary.pdf]

gels

MDPI

Supporting information

Highly Crosslinked Agar/Acrylic Acid Hydrogels with Antimicrobial Properties

Victor H. Pino-Ramos [1,*], Lorena Duarte-Peña [2] and Emilio Bucio [2]

[1] Departamento de Química Inorgánica y Nuclear, Facultad de Química, Universidad Nacional Autónoma de México, Avenida Universidad 3000, Ciudad Universitaria, Ciudad de México 04510, Mexico
[2] Departamento de Química de Radiaciones y Radioquímica, Instituto de Ciencias Nucleares, Universidad Nacional Autónoma de México, Circuito Exterior, Ciudad Universitaria, Ciudad de México 04510, Mexico; lorena.duarte@correo.nucleares.unam.mx (L.D.-P.); ebucio@nucleares.unam.mx (E.B.)
* Correspondence: Victor_Pino@comunidad.unam.mx

Citation: Pino-Ramos, V.H.; Duarte-Peña, L.; Bucio, E. Highly Crosslinked Agar/Acrylic acid Hydrogels with Antimicrobial Properties. *Gels* **2021**, *7*, 183. https://doi.org/10.3390/gels7040183

Academic Editor: Yi Cao

Received: 20 September 2021
Accepted: 22 October 2021
Published: 26 October 2021

Publisher's Note: MDPI stays neutral with regard to jurisdictional claims in published maps and institutional affiliations.

Copyright: © 2021 by the authors. Licensee MDPI, Basel, Switzerland. This article is an open access article distributed under the terms and conditions of the Creative Commons Attribution (CC BY) license (https://creativecommons.org/licenses/by/4.0/).

**File Name:**	AgNP.dts	**Dispersant Name:**	Water
**Record Number:**	10	**Dispersant RI:**	1.330
**Material RI:**	1.59	**Viscosity (cP):**	0.8872
**Material Absorbtion:**	0.01	**Measurement Date and Time:**	miércoles, 25 de agosto de 2...

System

**Temperature (°C):**	25.0	**Duration Used (s):**	60
**Count Rate (kcps):**	326.7	**Measurement Position (mm):**	1.25
**Cell Description:**	Disposable sizing cuvette	**Attenuator:**	5

Results

			**Diam. (nm)**	**% Intensity**	**Width (nm)**
**Z-Average (d.nm):**	49.7	**Peak 1:**	76.3	85.2	30.9
**PdI:**	0.443	**Peak 2:**	9.45	11.8	2.41
**Intercept:**	0.844	**Peak 3:**	4970	3.0	622

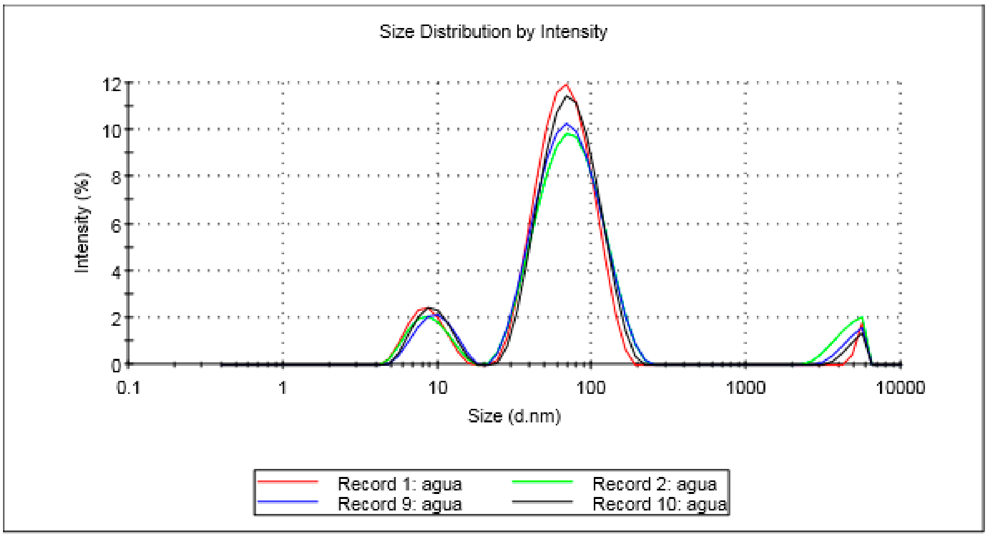

Figure S1. DLS measurements of silver nanoparticle suspension.
